# Keratocytes Derived from Spheroid Culture of Corneal Stromal Cells Resemble Tissue Resident Keratocytes

**DOI:** 10.1371/journal.pone.0112781

**Published:** 2014-11-10

**Authors:** Yong-Soo Byun, Sapna Tibrewal, Eunjae Kim, Lisette Yco, Joy Sarkar, Yair Ivanir, Chia-Yang Liu, Cecile M. Sano, Sandeep Jain

**Affiliations:** 1 Corneal Neurobiology Laboratory, Department of Ophthalmology and Visual Sciences, College of Medicine, University of Illinois at Chicago, Chicago, Illinois, United States of America; 2 Catholic Institute for Visual Science, Department of Ophthalmology and Visual Science, College of Medicine, Catholic University of Korea, Seoul, Korea; 3 Department of Molecular Biosciences and Bioengineering, University of Hawaii at Manoa, Honolulu, Hawaii, United States of America; 4 Department of Ophthalmology, College of Medicine, University of Cincinnati, Cincinnati, Ohio, United States of America; Instituto Butantan, Brazil

## Abstract

**Purpose:**

Corneal stromal cells transform to precursor cells in spheroid culture. We determined whether keratocytes derived from spheroid culture of murine corneal stromal cells resemble tissue resident keratocytes.

**Methods:**

Spheroid culture was performed by seeding dissociated stromal cells onto ultra-low attachment plates containing serum-free mesenchymal stem cell culture medium. Spheroids were characterized with phenotype specific markers and stemness transcription factor genes. Spheroids and adherent cells in culture were induced to differentiate to keratocytes using keratocyte induction medium (KIM) and compared with tissue resident keratocytes.

**Results:**

Stromal cells formed spheroids in ultra-low attachment plates, but not in polystyrene tissue culture dishes. Keratocan expression and abundance was significantly higher in spheroids as compared to adherent cells whereas alpha-smooth muscle actin (α-SMA) was significantly lower. As compared to adherent culture-derived cells, the expressions of keratocan, aldehyde dehydrogenase (ALDH3A1) and α-SMA in spheroid-derived cells approximated much more closely the levels of these genes in tissue resident keratocytes. Of the stemness genes, Nanog and Oct4 were upregulated in the spheroids.

**Conclusion:**

Stemness transcription factor genes are upregulated in spheroids. Keratocytes derived from spheroids resemble tissue resident keratocytes, thus increasing manifolds the quantity of these cells for in-vitro experiments.

## Introduction

Corneal stromal cells play an essential role in wound healing, angiogenesis, and nerve regeneration [1]–[6]. Although they are quiescent in naïve corneas; stromal cells (keratocytes) transform into wound healing phenotypes (fibroblasts and myofibroblasts) after injury [7], [8]. The molecular events that accompany this transformation are the biological basis of corneal wound healing. These events were determined primarily using mouse corneas [9], [10]. Although the use of a murine model to investigate corneal stromal cells has several advantages [1], [5], [11], [12], it is limited by the small amount of tissue available. Mouse corneas are small and thin and have an average diameter of 2.6 mm and thickness of 100 µm, two-thirds of which is stroma [13], [14]. Given the small dimensions of mouse corneas, primary cultures of keratocytes require an inordinately large number of corneas to be pooled in order to generate a sufficient quantity of cells for molecular analysis [15], [16]. Furthermore, the supplements required to stimulate cell proliferation transform keratocytes into wound healing phenotypes, yielding a culture populated by mixed stromal cell phenotypes [17]. Strategies to generate pure cultures of each stromal cell phenotype are essential to allow for the analysis of molecular events that accompany their transformation from quiescent to wound healing phenotypes. Immortalized corneal stromal cell lines may provide a large amount of tissue, but being transfected, the molecular processes within these cells may not truly represent a physiological state of primary cells [18].

One strategy to generate pure cultures is via spheroid culture, a technique originally developed for obtaining multipotent neural crest-derived stem cells from corneal stromal cells [19]–[22]. Although it is known that spheroids derived from corneal stromal cells express stem cell markers [19], [23], [24], it is unclear whether they also express some or all of the stemness transcription factor genes. Differentiated cells can be reprogrammed to an embryonic-like state by increasing the expression of a few select transcription factors, namely *Oct3/4*, *Sox2*, *c-Myc*, and *Klf4* [25]. We hypothesize that the stemness transcription factor genes responsible for reprogramming differentiated cells are overexpressed during spheroidal culture of corneal stromal cells. In this study, we demonstrate that spheroids formed from murine corneal stromal cells had increased expression of several stemness transcription factor genes as compared to adherent cells and these spheroids can be induced to yield keratocytes in sufficient quantities.

## Methods

### Ethics Statement

All animal experiments were conducted in strict accordance with the recommendations in the Guide for the Care and Use of Laboratory Animals of the National Institutes of Health. The protocol was approved by the Institutional Animal Care and Use Committee (IACUC) of the University of Illinois at Chicago (Protocol Number: 13-159). *Thy1*-YFP neurofluorescent homozygous adult mice (6–8 weeks old) were purchased from Jackson Laboratories (Bar Harbor, ME), and colonies were established by inbreeding. Mice were sacrificed for terminal experiments according to the IACUC protocol. All efforts were made to minimize suffering.

### Isolation and Culture of Primary Corneal Stromal Cells

*Thy1*-YFP mice (6 to 8 week-old) were sacrificed and corneal stromal cells were isolated using a modified three sequential collagenase digestion protocol, as previously described [17]. Corneal disks were excised using sterile corneal scissors and forceps. The disks were placed in a microcentrifuge tube containing 1 ml of 3 mg/mL collagenase (Sigma-Aldrich, St. Louis, MO) prepared in Dulbecco’s Modified Eagle’s Medium F12 (DMEM/F12; Life Technologies, Grand Island, NY). The corneas were digested at 37°C for 30 minutes with shaking. The digested contents were then filtered through a 70 µm nylon cell strainer (BD Biosciences, Bedford, MA), and the tissue pieces collected were placed in a new microcentrifuge tube containing 1 ml of fresh 3 mg/ml collagenase in DMEM/F12 and digested at 37°C with shaking for 60 minutes. The remaining tissue pieces were again collected, added to fresh collagenase in DMEM/F12, and digested at 37°C with shaking for 180 minutes. Following the last digestion, tissue pieces were vortexed for 30 seconds, strained and discarded from the collagenase solution. The cells in the collagenase solution were then collected by low-speed centrifugation (1400 rpm for 5 minutes). Cells were resuspended in DMEM/F12 supplemented with 10% fetal bovine serum (FBS; Life Technologies) and antibiotic-antimycotic solution (100 IU/ml penicillin, 100 mg/ml streptomycin, 2.5 µg/ml amphotericin B; Mediatech, Manassas, VA). Resuspended cells were used directly for experiments (as specified later in the text) or plated in 35-mm poly-d-lysine (PDL; Sigma-Aldrich) coated tissue culture dishes and incubated at 37°C in a humidified atmosphere at 5% CO_2_. After initial primary culture, adherent cells were expanded in 15-cm PDL coated tissue culture dishes. Medium was changed every 3 days. Some cells were stained with epithelial keratin to exclude any contamination by epithelial cells.

### Spheroidal Culture of Corneal Stromal Cells

After establishing and expanding adherent cultures, cells were trypsinized (0.25% Trypsin-EDTA (1x), phenol red; Life Technologies) and were seeded (2×10^4^ cells/well) in 6-well ultra-low attachment (ULA) plates (Corning Inc, Corning, NY) in StemPro mesenchymal stem cell serum free medium (Life Technologies). The cells were incubated at 37°C in a humidified atmosphere at 5% CO_2_ for 4–5 days to form spheroids.

### Differentiation of adherent cells and spheroids to stromal cell phenotypes

Three different conditional media were used to induce differentiation of adherent and spheroid cells to keratocytes, fibroblasts, and myofibroblasts. Briefly, equivalent passaged (P6) adherent and spheroid cells were seeded (2×10^4^ cells/cm^2^) in keratocyte induction medium (KIM) consisting of serum-free DMEM/F12 with 0.1 mM L-Ascorbic acid (Sigma-Aldrich) for keratocyte induction. Fibroblasts were induced using fibroblast induction medium (FIM) consisting of DMEM/F12 with 10% FBS and 10 ng/ml recombinant human basic fibroblast growth factor (bFGF; R&D Systems, Minneapolis, MN) [26], [27]. Finally, differentiation to myofibroblasts was induced using myofibroblast induction media (MIM) consisting of DMEM/F12 with 10% FBS and 1 ng/ml recombinant human transforming growth factor (TGF-β 1; R&D Systems) [26]–[28]. Cells were stained with 1 µM Calcein acetoxymethylester (Calcein AM, BD Biosciences), a fluorescent dye for live cells. For quantitative viability assessment, cells were trypsinized, and the number of live cells was counted using an automated cell counter.

### Western Blot Analysis

Cultured cells were lysed in a radioimmunoprecipitation assay (RIPA) buffer (20 mM Tris-HCl, pH 7.5, 0.1% sodium lauryl sulfate, 0.5% sodium deoxycholate, 135 mM NaCl, 1% Triton X-100, 10% glycerol, 2 mM EDTA) supplemented with complete protease inhibitor and phosphatase inhibitor cocktails I and II (Sigma-Aldrich). Cell lysate was centrifuged at 14,000 rpm for 10 minutes and the supernatant was used. Total protein concentration was determined using Bradford dye reagent protein assay (Bio-Rad Laboratories, Richmond, CA). Laemmli sample buffer (4x, Bio-Rad) containing 10% (v/v) β-mercaptoethanol was added to the cell lysate (20 µg) and boiled for 5 minutes. Samples were loaded on Novex Tris-Glycine mini gels (Life Technologies) for electrophoresis and then blotted onto a nitrocellulose membrane (Life Technologies). The membrane was placed in blocking buffer (5% nonfat dry milk in 1x Tris-buffered solution with 0.1% Tween-20; 0.1% T-TBS) for 1 hour at room temperature with shaking, and then incubated with primary antibodies (made in blocking buffer) overnight at 4°C with shaking. Following incubation, the membrane was washed three times with 0.1% T-TBS, and incubated with secondary antibodies in 0.1% T-TBS for 1 hour at room temperature with shaking. After three washes with 0.1% T-TBS, bands were detected using the Odyssey Infrared Imaging System (Li-Cor Biosciences, Lincoln, NE). NIH Image J 1.44 software was used to calculate the intensity of the bands. Protein bands were normalized relative to β-actin and GAPDH. The primary antibodies used were goat polyclonal anti-keratocan C (KERA; 1∶500; generously provided by Dr. Chia-Yang Liu, University of Cincinnati, Cincinnati, OH), mouse monoclonal anti-actin, α-smooth muscle (α-SMA; 1∶500, a2547, Sigma-Aldrich), rabbit polyclonal anti-aldehyde dehydrogenase family 3 member A1 (ALDH3A1; 1∶500, ab76976, Abcam), rabbit polyclonal anti-β-actin (1∶2000; Cell Signaling, Danvers, MA), and mouse monoclonal anti-GAPDH (1∶500, Santa Cruz Biotechnology, Santa Cruz, CA). We used goat anti-mouse (IRDye 800DX, 1∶10,000), donkey anti-goat (IRDye 800CW, 1∶10,000), and goat anti-rabbit (IRDye 680CW, 1∶10,000) secondary antibodies (Rockland Immunoresearch, Gilbertsville, PA).

### RNA Preparation and Real-time quantitative PCR analysis

Total RNA was extracted from each cell line using a commercial reagent (TRIzol reagent; Life Technologies) according to manufacturer’s instructions. The concentration of the total RNA was determined using a spectrophotometer (NanoDrop 1000, Thermo Scientific). After purifying samples using a TURBO DNA-free Kit (Ambion, Life Technologies), 2 µg RNA was used for cDNA synthesis using a High Capacity cDNA Reverse Transcription Kit (Applied Biosystems, Foster City, CA). Signals were detected with a 7900 HT Real-Time PCR System (Applied Biosystems). The following primers were used: Integrin alpha-6 (CD49f; Forward: 5- GTGGCCCAAGGAGATTAGC-3, Reverse: 5- GTTGACGCTGCAGTTGAGA-3) [29], octamer-binding transcription factor 4 (Oct4; cat #PPM04726; Qiagen Inc., Valencia, CA), Nanog homeobox (Nanog; cat# PPM 25326B), kruppel-like factor 4 (Klf4; cat #PPM25088A), myelocytomatosis oncogene (c-Myc; cat #PPM02924E), Keratocan (KERA; Forward: 5-CTGAGGCTCAACCACAACAA-3, Reverse: 5-GTGCTGCAGGTTAGCATTGA-3) [30], aldehyde dehydrogenase family 3, member A1 (ALDH3A1; cat #PPM25079A), alpha-smooth muscle actin (α-SMA; cat #PPM04483A), and glyceraldehyde-3-phosphate dehydrogenase (GAPDH; cat #PPM02946E). The comparative threshold cycle (2^−ΔCT^) method was used to determine mRNA expression level of each gene as compared to that of GAPDH. The fold change of mRNA expression between adherent cells and spheroids was calculated, using the formula 2^−ΔΔCT^.

### Immunofluorescence Microscopy

For immunostaining, cells were washed twice with 1x phosphate buffered saline (PBS), fixed with 4% formaldehyde for 15 minutes, and permeabilized in 0.1% Triton X-100 for 10 minutes at room temperature. Fixed cells were washed with PBS, blocked in 0.1% Tween-20 (T-PBS) with 2% donkey serum for 1 hour at room temperature, and then incubated with primary antibody overnight at 4°C. The cells were then washed three times with 0.1% T-PBS, followed by one wash with PBS. The cells were incubated with the corresponding secondary antibody (Jackson ImmunoResearch Lab, West Grove, PA) in 0.1% T-PBS for 1 hour at room temperature. Finally, cells were washed three times with 0.1% T-PBS and once with distilled water. For the spheroids, modified permeabilizing and mounting method was used to help antibodies penetrate inside and preserve their 3D structure [31]. Spheroids in suspension were fixed and permeabilized in PBS containing 4% formaldehyde and 1% Triton X-100 at 4°C for 3 h, and washed three times with PBS. Spheroids were then dehydrated in an ascending series of methanol in PBS (25%, 50%, 75%, 95%, 30 min each and 100% for 5 h) at 4°C, rehydrated in the reverse descending series and washed three times with PBS. Mounting was carried out in a simple chamber assembled from a glass slide and a cover slip, using double-sided scotch as ‘spacer’ between them. Spheroids were resuspended in a drop of DAPI-containing mounting medium and mounted in the space between a glass slide and a cover slip. The primary antibodies used were mouse monoclonal anti-Oct4 (1∶200; MAB4305, Millipore), rabbit polyclonal anti-Nanog (1∶200; ab80892, Abcam), goat polyclonal anti-KERA (1∶200), rabbit polyclonal anti-ALDH3A1 (1∶200), and mouse monoclonal anti- α-SMA (1∶400). Images of spheroid were taken at an interval of 2 µm from top to the bottom to confirm if antibodies penetrated regardless of the depth. Immunofluorescence images were obtained using a Zeiss Axio Observer. Z1 inverted microscope and LSM 710 confocal microscope (Carl Zeiss, GmbH, Hamburg, Germany).

### Statistical Analysis

Following compilation of data using Microsoft Excel office statistics software (Redmund, WA), the arithmetic means and standard errors (SEs) of means were calculated for all quantitative parameters. Analysis of variance (ANOVA) was used to compare mean values between groups (SPSS Statistics, version 22; IBM Corporation, Armonk, NY). *P*≤0.05 was considered statistically significant.

## Results

### Characteristics of adherent cells and spheroids

**Figure 1:**
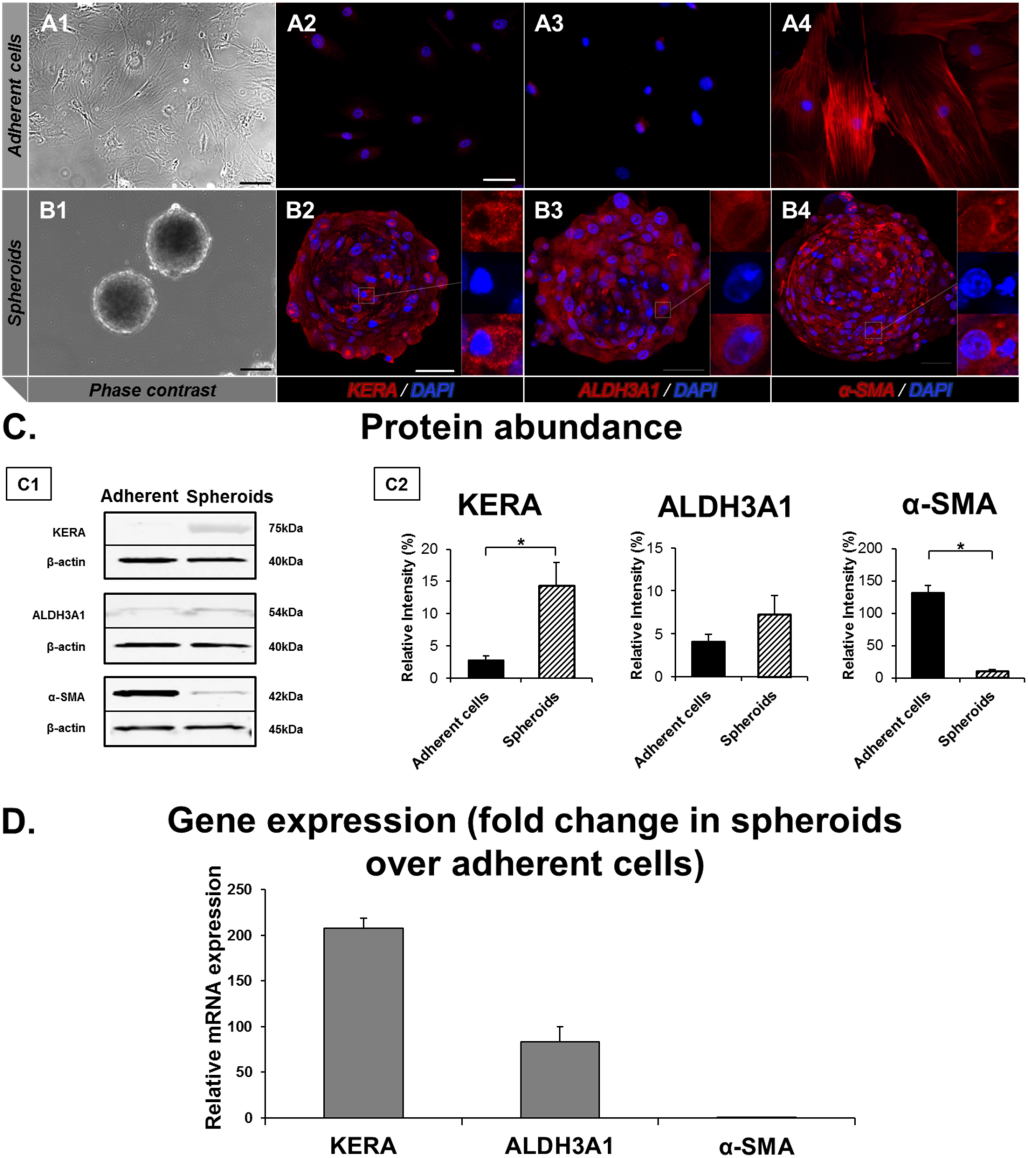
Characteristics of adherent cells and spheroids. (**A1**) Phase contrast image showing adherent stromal cells in serum-based growth media. (**B1**) Phase contrast image of spheroids in ultra-low attachment plates with serum-free media. (**A2–4**) On immunofluorescence staining, adherent cells stain weakly with KERA (**A2**, red) and ALDH3A1 (**A3**, red), and are strongly positive for α-SMA (**A4**, red). (**B2–4**) In contrast, spheroids stain strongly positive for KERA (**B2,** red), ALDH3A1 (**B3,** red), as well as α-SMA (**B4,** red). Cell nuclei are stained with DAPI (blue). (**C**) Western blot results for protein abundance in adherent cells and spheroids. Representative blot image (**C1**) and graph (**C2**) show that KERA and ALDH3A1 are significantly more abundant in spheroids, while α-SMA is more abundant in adherent cells. β-Actin was used as a loading control. (**D**) RT-qPCR confirms KERA and ALDH3A1 gene expressions to be higher in spheroids than in adherent cells, while α-SMA is expressed more in adherent cells than in spheroids. KERA, keratocan; ALDH3A1, aldehyde dehydrogenase 3 family member A1; α-SMA, alpha-smooth muscle actin. DAPI, 4′,6-diamidino-2-phenylindole. Scale bars: 50µm; Error bars = Standard error of mean; *p≤0.05.

We performed immunofluorescence staining, Western blot, and real time quantitative PCR (RT-qPCR) to study the morphology and gene expression profiles of adherent cells and spheroids (Fig. 1). On light microscopy, adherent stromal cells were spindle shaped, consistent with activated phenotypes (fibroblasts and myofibroblasts; Fig. 1A1). In ultra-low attachment plates, cells coalesced to form spheroids within 2 days (Fig. 1B1). On immunofluorescence, cultured adherent cells stained weakly with KERA and ALDH3A1, but were strongly positive for α-SMA (Figs. 1A2–A4), while spheroids stained strongly positive for KERA and ALDH3A1, as well as α-SMA (Figs. 1B2–B4). Western blot analysis showed that KERA was significantly more abundant in spheroids than in adherent cells (14.3±3.6 and 2.8±0.7, respectively; *P*≤0.05). ALDH3A1 was also more abundant in spheroids than in adherent cells (7.3±4.4 and 4.1±0.9, respectively), but was short of statistical significance. The abundance of α-SMA was significantly lower in spheroids than in adherent cells (10.3±2.5 and 131.9±11.1, respectively; *P*≤0.05; Figs. 1C1–C2). RT-qPCR confirmed that KERA and ALDH3A1 were significantly more expressed (207.4±10.8 and 82.8±16.9 folds, respectively), while α-SMA was less expressed (0.02±0.09 fold) in the spheroids, as compared to adherent cells (*P*≤0.05; Fig. 1D). Collectively, these results suggest that primary stromal cells grown in spheroid culture conditions express molecules that are typically expressed by keratocytes.

### Forced differentiation of adherent cells and spheroids

**Figure 2:**
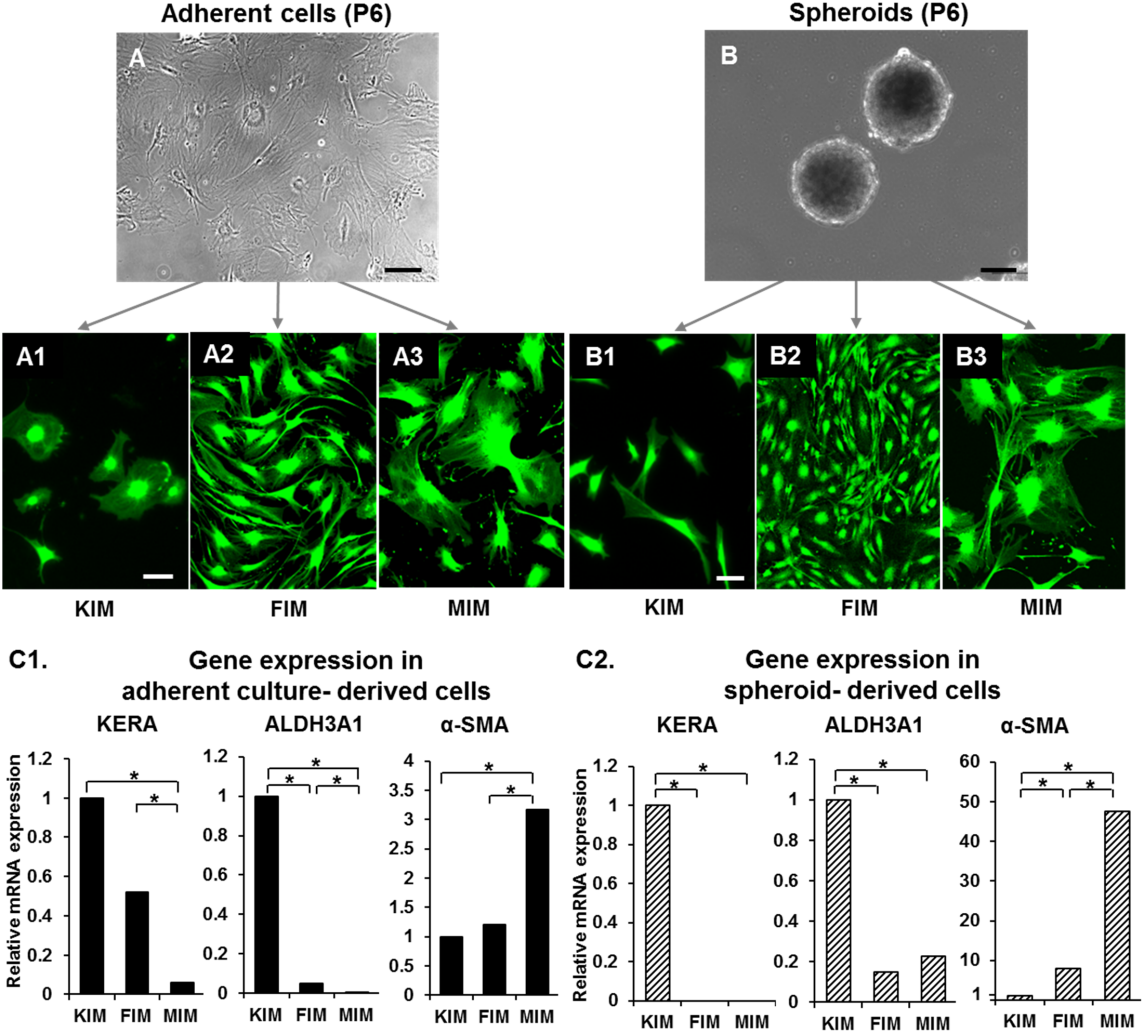
Forced differentiation of adherent cells and spheroids. Calcein AM stained images show forced induction of adherent cells (**A1–3**) and spheroids (**B1–3**) in KIM, FIM, and MIM. (**C1, C2**) Graphs show relative gene expression of KERA, ALDH3A1, and α-SMA in adherent culture-derived and spheroid-derived cells in KIM, FIM, and MIM. (**C1**) In adherent culture-derived cells, KERA and ALDH3A1 expression is highest in KIM, while α-SMA expression is highest in MIM. **(C2)** In spheroid-derived cells also, the highest expression of KERA and ALDH3A1 is seen in KIM. KIM, keratocyte induction medium; FIM, fibroblast induction medium; MIM, myofibroblast induction medium; bFGF, basic fibroblast growth factor; TGF-β, transforming growth factor β; KERA, keratocan; ALDH3A1, aldehyde dehydrogenase 3 family member A1; α-SMA, alpha-smooth muscle actin. Scale bars: 50 µm; *p≤0.05.

To assess if spheroids can be induced into stromal cell phenotypes, we cultured spheroids and adherent cells in KIM, FIM, and MIM to induce differentiation to keratocytes, fibroblasts, and myofibroblasts, respectively. Very few of adherent culture-derived cells in KIM showed morphological features of keratocytes (Fig. 2A1); whereas majority of spheroid-derived cells in KIM exhibited dendritic processes; a morphological feature consistent with keratocytes (Fig. 2B1). Adherent culture-derived as well as spheroid-derived cells in FIM and MIM demonstrated morphological features similar to fibroblasts and myofibroblasts; respectively (Figs. 2A2–3 and figs. 2B2–3).

To study the gene expression profile of adherent culture-derived and spheroid-derived cells in KIM, FIM and MIM, we performed RT-qPCR for KERA, ALDH3A1, and α-SMA expressions (Figs. 2C1–C2). KERA and ALDH3A1 were most highly expressed in adherent cells and spheroids grown in KIM, as compared to those grown in MIM, or FIM, while α-SMA was most highly expressed in adherent cells and spheroids grown in MIM, as compared to those grown in KIM or FIM. We compared the expression levels of KERA, ALDH3A1, and α-SMA between spheroid-derived and adherent culture-derived cells. Spheroid-derived cells in KIM showed significantly higher expression levels of KERA and ALDH3A1 (37.15±2.91 and 1.33±0.14 folds, respectively) and lower expression of α-SMA (0.15±0.16 fold) than adherent culture-derived cells in KIM (*P*≤0.05). There was no significant difference in the expression levels of stromal phenotype markers between adherent culture-derived and spheroid-derived cells in FIM or MIM.

**Figure 3:**
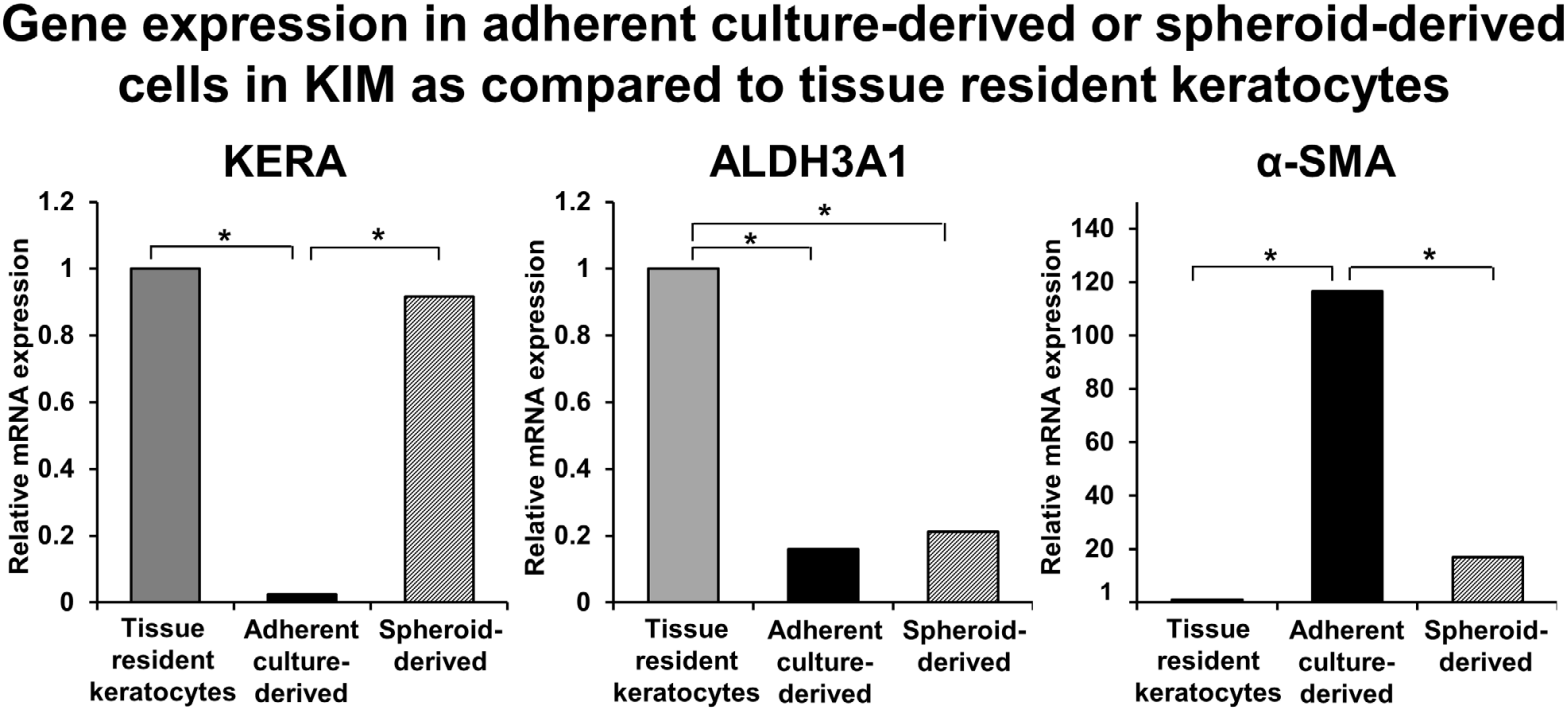
Graph shows that KERA and α-SMA expressions in spheroid-derived cells are similar to those in tissue resident keratocytes. Adherent culture-derived cells have significantly decreased KERA expression and increased α-SMA expression, as compared to spheroid-derived cells and tissue resident keratocytes. KIM: keratocyte induction medium; KERA: keratocan; ALDH3A1: aldehyde dehydrogenase 3 family member A1; α-SMA: alpha-smooth muscle actin. *p≤0.05.

We also compared the expression levels of phenotype specific markers in adherent culture-derived and spheroid-derived cells in KIM, with tissue resident keratocytes obtained directly from corneal stromal lysate after triple digestion. As compared to adherent culture-derived cells, the expression of KERA, ALDH3A1 and α-SMA in spheroid-derived cells approximated much more closely the levels of these genes in tissue resident keratocytes (Fig. 3). KERA expression was significantly reduced in adherent culture-derived cells, as compared to spheroid-derived cells and tissue resident keratocytes (0.013±0.006, 0.486±0.16, and 0.530±0.26, respectively; *P*≤0.05). α-SMA expression was significantly increased in adherent culture-derived cells, as compared to spheroid-derived cells and tissue resident keratocytes (0.068±0.009, 0.01±0.003, and 0.0006±0.0003, respectively; *P*≤0.05). These results suggest that spheroid-derived cells in KIM express phenotype specific markers and thus resemble tissue resident keratocytes more closely than adherent culture-derived cells in KIM.

### Morphology and viability of keratocytes derived from adherent cells versus spheroids

**Figure 4:**
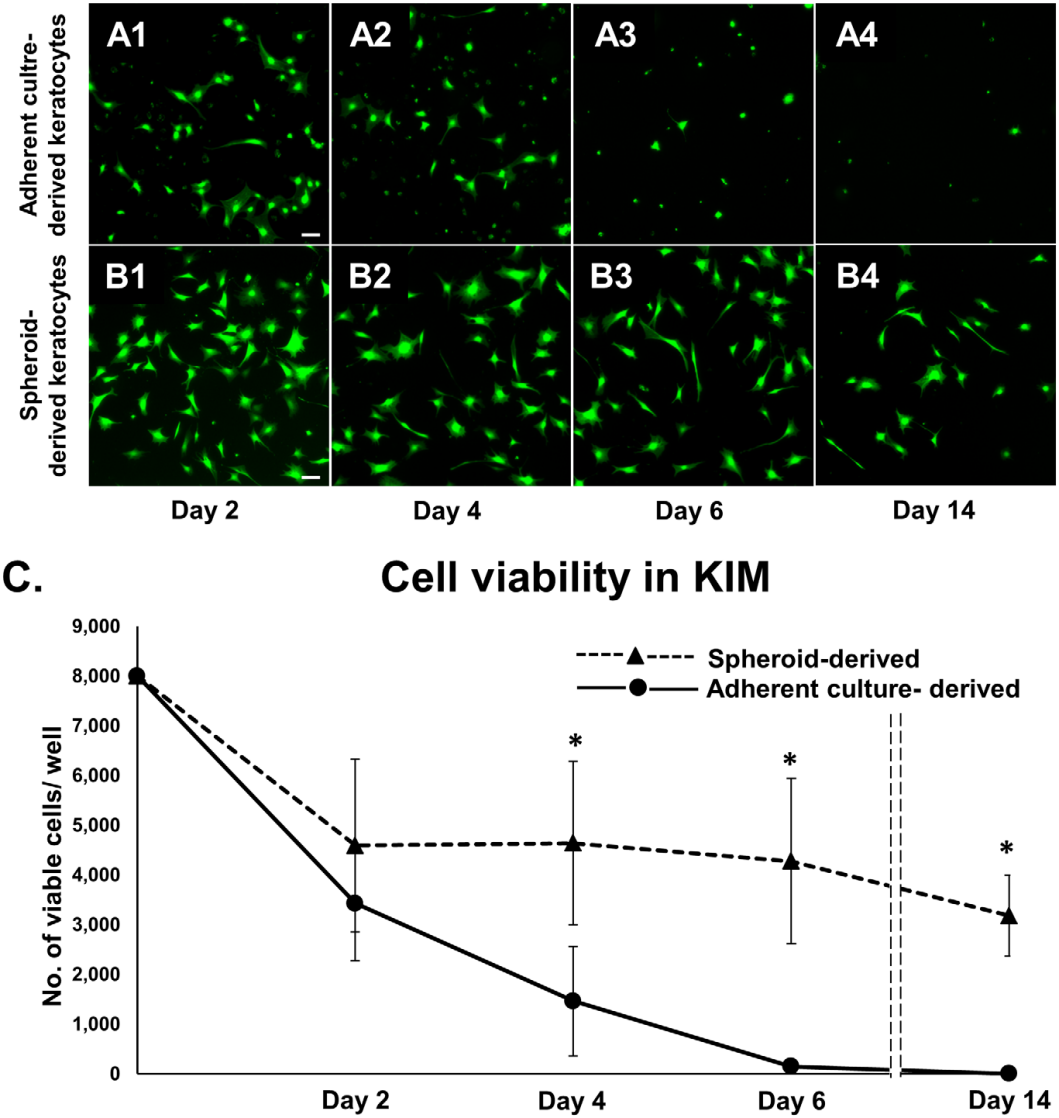
Spheroid-derived cells in KIM resemble keratocytes and are more viable than adherent culture-derived cells. (**A1–4**) Wide field fluorescence images of Calcein AM stained cells show very few dendritic processes in adherent culture-derived cells in KIM. (**B1–4**) In contrast, spheroid-derived cells show typical keratocyte morphology with dendritic processes and stellate shape, and this is maintained over 2 weeks of culture. (**C**) Cell viability analysis shows that keratocytes derived from spheroids were more viable than those derived from adherent cells over 2 weeks. Error bars = Standard error of mean; Scale bars: 100 µm; *p≤0.05.

**Figure 5:**
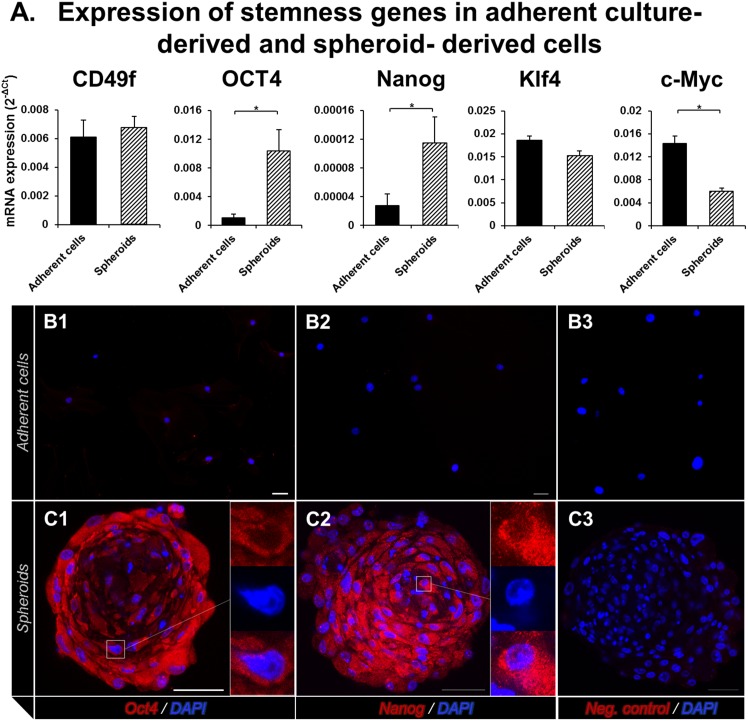
Expression of stemness transcription factor genes in spheroids and adherent cells. (**A**) RT-qPCR analyses shows that Oct4, and Nanog are expressed significantly more in spheroids than in adherent cells. (**B and C**) Immunofluorescence stained images show that adherent cells do not stain with Oct4 (**B1**, red) and Nanog (**B2**, red), whereas spheroids show positive staining with both (**C1, C2**). Nuclei stain with DAPI (blue). (**B3, C3**) Negative controls. Oct4, octamer-binding transcription factor 4; Nanog, Nanog homeobox; DAPI, 4′,6-diamidino-2-phenylindole. Error bars = Standard error of mean; Scale bars: 50 µm; *p≤0.05.

To evaluate the morphology and viability of cells during keratocyte induction, adherent culture-derived and spheroid-derived cells were seeded in 12-well plates (8×10^3^ cells/well) with serum free KIM. On days 2, 4, 6 and 14 after seeding, cells were stained with Calcein AM for morphology and viability assessment (Fig. 4). Adherent culture-derived cells in KIM showed very few dendritic processes (Figs. 4A1–A4). In contrast, spheroid-derived cells in KIM showed typical keratocyte morphology with dendritic processes and stellate shape (Figs. 4B1–B4). As compared to viability of cells on day 0 (100%), the viability of adherent culture-derived as well as spheroid-derived cells decreased significantly on day 2 (42.8±8.3% and 57.4±21.6%, respectively; *P*<0.05). After 2 days, the viability of adherent culture-derived cells continued to reduce significantly to 18.3±7.9% on day 4, 1.8±0.8% on day 6, and 0.02±0.01% on day 14 (*P*<0.05 as compared to day 2). In contrast, the viability of spheroid-derived cells remained unchanged on day 4 (57.9±11.9%) and day 6 (53.4±12.0%; *P*>0.05). On day 14, viability reduced significantly to 39.8±5.9% (*P*<0.05). The viability of spheroid-derived cells was significantly higher than that of adherent culture-derived cells on days 4, 6, and 14 (*P*<0.05; Fig. 5C).

### Expression of stemness transcription factor genes in spheroids

To determine if the spheroids express stemness transcription factor genes, we performed RT-PCR for *CD49f*, *Oct4*, *Nanog*, *Klf4*, and *c-Myc* genes (Fig. 5A). The expressions of *Oct4* and *Nanog* were significantly higher in spheroids than in adherent cells (10.33 and 4.15 folds, respectively; *P*≤0.05). There was no significant difference in the expressions of *CD49f*, *Klf4*, and *c-Myc* between the spheroids and adherent cells.

To further confirm the increased expression of the two upregulated genes (*Oct4* and *Nanog*) and to localize the proteins in spheroids, we performed immunofluorescence staining for *Oct4* and *Nanog* in adherent cells and spheroids (Figs. 5B and C). Unlike no staining in adherent cells (Figs. 5B1–B2), spheroids stained positive for *Oct4* and *Nanog* (Figs. 5C1–C2). In the spheroids, both *Oct4* and *Nanog* localized to the cytoplasm.

## Discussion

In this study, we used the spheroid culture method to increase the yield of keratocytes obtained after three sequential collagenase digestion of murine corneas. We showed that spheroid-derived cells, when induced to form keratocytes in KIM, express markers that closely resemble the expression profile of tissue resident keratocytes. In the normal cornea, keratocytes have high levels of ALDH3A1 and low levels of α-SMA [32]–[34]. This expected pattern was absent during the induced differentiation of dissociated cells derived from adherent cell culture, but it was achieved by forced differentiation of dissociated cells derived from spheroids. We also showed that the spheroid-derived keratocytes were more viable than adherent culture-derived cells. Lastly, we showed that the spheroids express stemness transcription factor genes, which can explain, at least in part, their potential to de-differentiate terminally differentiated cells into precursor cells under appropriate culture conditions. Thus, we show that the spheroid culture method can be used to increase the yield of murine primary keratocyte populations for in-vitro experiments.

Our findings build upon those reported by Yoshida et al [20]. They too used the spheroidal culture method to yield larger quantities of primary murine keratocytes. In contrast to Yoshida et al, we have isolated primary keratocytes using three sequential collagenase digestion and cultured spheroids in ultra- low attachment plates. The three sequential collagenase digestion of stromal tissue has been shown to be helpful in isolating keratocytes in their native phenotype [17]. This method avoids keratocyte apoptosis that may be induced by epithelial scraping [35], [36]. The stromal cells released during the first collagenase digestion represent epithelial cells, and those in the second digestion are damaged keratocytes with poor viability and attachment properties [17]. We did not perform explant cultures to isolate stromal cells. In full thickness corneal explant cultures the epithelium extends along the cut edges and few, if any, stromal cells migrate out [37]. Scott et al have also demonstrated spheroid formation when dissociated human and bovine corneal cells were seeded onto ultra-low attachment culture dishes [38]. In that study, keratocytes were seeded for spheroidal culture. In contrast, our study involved seeding a mixed population of fibroblasts and myofibroblasts, which represent terminally differentiated cells. We also used a proprietary serum-free mesenchymal stem cell culture medium (StemPro) for the spheroidal culture. This culture medium allows for expansion of mesenchymal stem cells while maintaining their multipotent phenotype by controlling transforming growth factor (TGF)–β, platelet-derived growth factor (PDGF), and fibroblast growth factor (FGF) signaling pathways [39], [40]. Interestingly, spheroid formation occurs in other cell culture media as well. Scott et al [38] report spheroid formation in keratocyte plating medium (KPM) consisting of serum-free DMEM/F12, keratocyte growth medium (KGM) consisting of DMEM/F12 with FGF2, and fibroblast growth medium (FGM) consisting of DMEM/F12 with FBS. Spheroids grown in KGM were the largest at approximately 160 µm in diameter. We also observed large, approximately 200 µm diameter spheroids in StemPro cell culture medium.

Our data showed that mRNA expression as well as protein abundance of ALDH3A1 was higher in spheroids as compared to adherent cells. However, there is discordance between the extent of mRNA expression and protein abundance of ALDH3A1. In their review, Greenbaum et al [41] have identified at least three reasons for the poor correlations between the level of mRNA and the level of protein, and these may not be mutually exclusive. First, there are many complicated and varied post-transcriptional mechanisms involved in turning mRNA into protein that are not yet sufficiently well-defined to be able to compute protein concentrations from mRNA. Posttranslational modifications are well known in the case of ALDH family [42]; second, protein turnover can vary significantly depending on a number of different conditions. Cells can control the rates of degradation or synthesis of a protein, thus affecting its abundance; and/or third, there is a significant amount of error and noise in both protein and mRNA experiments that limit our ability to get a clear picture of the correlation between their levels [41]. Any or all of these three possibilities may explain our data. To be fully able to understand the reasons for this discordance, the dynamic processes involved in synthesis and degradation of ALDH3A1 will have to be investigated in future studies.

We showed that select stemness transcription factor genes are expressed when adherent corneal stromal cells transform to spheroids in ultra-low attachment plates. We observed upregulation of *Oct4* and *Nanog* genes in spheroids. We did not assay freshly isolated keratocytes for stemness factors. However, Chien et al [43] have performed RT-PCR and showed that stemness genes (Oct4, Sox2 and Nanog) are not expressed in keratocytes. Furthermore, other stem cell markers like Musashi-1 have been shown to be expressed in spheroids but not in keratocytes [19]. Viewed together with these published data, our data suggests that enhanced expression of stemness genes may have reprogrammed the differentiated stromal cells in adherent culture to an earlier precursor form in spheroids. Classically, reprogramming of differentiated cells is induced by intracellular introduction of essential reprogramming genes by transfection [25], [44], [45]. Murine fibroblasts can yield pluripotent stem cells following introduction of ES cell markers like *Oct3/4*, *Sox2*, *c-Myc*, and *Klf4* [25]. Global gene expression patterns and changes in DNA methylation confirm that such induced pluripotent cells are not ES cells. This lends credence to the hypothesis that differentiated cells could be “de-differentiated” under the appropriate conditions. In the context of cornea, such plasticity has been demonstrated in cultured stromal cells. Culture of keratocytes in fibroblast growth medium transforms them into the fibroblast phenotype and removal of growth factor support leads to a reversal back to a keratocyte phenotype [46]. In our experiments, reprogramming of stromal cell phenotypes to an earlier precursor form was achieved by transferring the cells from adherent to non-adherent plates containing stem cell culture medium. Therefore, our data suggests that changes in the physical condition of the cell, combined with stem cell culture medium, can stimulate inherent plasticity in differentiated cells, albeit in a limited fashion. Our data agrees with reports that suggest that stem cell fate can be influenced by controlling cell shape and the surrounding extracellular matrices. For example, cell shape (i.e., rounded versus flattened morphology) controls the lineage commitment of mesenchymal stem cells into adipogenic or osteoblastic phenotypes [47]. The most direct and practical implication of our study is that it provides a method of generating pure cultures of corneal stromal cell phenotypes (keratocytes, fibroblasts, and myofibroblasts) without using an inordinately large number of animals. Wound healing phenotypes of stromal cells (fibroblasts and myofibroblasts) are the structural basis of haze and scar, and our data demonstrate the experimental feasibility of reverting these phenotypes. Our data suggests that keratocytes, fibroblasts, and myofibroblasts may possess inherent plasticity that can be activated. Therefore, it may be possible to influence corneal clarity by developing cell reprogramming strategies. This could replace the current treatment of clearing corneal scars by topical application of Mitomycin C, which induces apoptosis in myofibroblasts and inhibits fibroblast proliferation [48].

In conclusion, our study suggests that it is possible to obtain keratocyte, fibroblast, and myofibroblast populations from spheroids, providing a multifold increase in the number of primary murine stromal cells. This reduces the need for inordinately large numbers of corneas. Our findings also suggest that it is possible to transform terminally differentiated corneal stromal cell phenotypes into precursor cells in-vitro.
